# Kufor-Rakeb syndrome-associated psychosis: a novel loss-of-function *ATP13A2* variant and response to antipsychotic therapy

**DOI:** 10.1007/s10048-024-00767-7

**Published:** 2024-07-18

**Authors:** Mark Ainsley Colijn, Stephanie Vrijsen, Ping Yee Billie Au, Rania Abou El Asrar, Marine Houdou, Chris Van den Haute, Justyna Sarna, Greg Montgomery, Peter Vangheluwe

**Affiliations:** 1 Department of Psychiatry, University of Calgary, Calgary, AB Canada; 2 Mathison Centre for Mental Health Research and Education, University of Calgary, Calgary, AB Canada; 3 Hotchkiss Brain Institute, University of Calgary, Calgary, AB Canada; 4 Department of Medical Genetics, Cumming School of Medicine, Alberta Children’s Hospital Research Institute, University of Calgary, Calgary, AB Canada; 5 Department of Clinical Neurosciences, University of Calgary, Calgary, AB Canada; 6 Laboratory of Cellular Transport Systems, Department of Cellular and Molecular Medicine, KU Leuven, Leuven, B-3000 Belgium; 7 Aligning Science Across Parkinson’s (ASAP) Collaborative Research Network , Chevy Chase, MD 20815 USA; 8 Leuven Viral Vector Core KU Leuven, Leuven, B-3000 Belgium; 9 Research Group for Neurobiology and Gene Therapy, Department of Neurosciences, KU Leuven, Leuven, B-3000 Belgium

**Keywords:** Antipsychotic agents, Genetics, behavioral, Quetiapine fumarate, Neuropsychiatry, Psychotic disorders

## Abstract

Biallelic (autosomal recessive) pathogenic variants in *ATP13A2* cause a form of juvenile-onset parkinsonism, termed Kufor-Rakeb syndrome. In addition to motor symptoms, a variety of other neurological and psychiatric symptoms may occur in affected individuals, including supranuclear gaze palsy and cognitive decline. Although psychotic symptoms are often reported, response to antipsychotic therapy is not well described in previous case reports/series. As such, we describe treatment response in an individual with Kufor-Rakeb syndrome-associated psychosis. His disease was caused by a homozygous novel loss-of-function *ATP13A2* variant (NM_022089.4, c.1970_1975del) that was characterized in this study. Our patient exhibited a good response to quetiapine monotherapy, which he has so far tolerated well. We also reviewed the literature and summarized all previous descriptions of antipsychotic treatment response. Although its use has infrequently been described in Kufor-Rakeb syndrome, quetiapine is commonly used in other degenerative parkinsonian disorders, given its lower propensity to cause extrapyramidal symptoms. As such, quetiapine should be considered in the treatment of Kufor-Rakeb syndrome-associated psychosis when antipsychotic therapy is deemed necessary.

**Supplementary Information:**

The online version contains supplementary material available at 10.1007/s10048-024-00767-7.

## Introduction

Biallelic pathogenic variants in *ATP13A2*, also known as *PARK9*, are associated with an autosomal recessive form of juvenile-onset parkinsonism, termed Kufor-Rakeb syndrome (KRS). Additional clinical features include facial-faucial-finger myoclonus, supranuclear gaze palsy, oculogyric dystonic spasms, and dementia, in addition to various neuropsychiatric symptoms, including psychosis [1, 2]. Some affected individuals are considered to have a form of neurodegeneration with brain iron accumulation or neuronal ceroid lipofuscinosis [1, 2], and spastic paraplegia has also been described in relation to recessive *ATP13A2* variants [3].

*ATP13A2* encodes for the ATPase 13A2 (ATP13A2). ATP13A2 belongs to the superfamily of P-type ATPase transporters, which are alternatingly auto-phosphorylated and dephosphorylated on a conserved aspartic acid during their catalytic cycle [4]. ATP13A2 has recently been described as a polyamine transporter with highest affinity for the polyamines spermine and spermidine. At the cellular level, ATP13A2 is localized in late endolysosomes, where it exports the endocytosed polyamines spermine and spermidine from the lumen to the cytosol [5]. Polyamines are neuroprotective agents and are involved in a plethora of pathways, ranging from the regulation of transcription and translation to autophagy and anti-oxidant responses [6]. As ion channel modulators, polyamines have been implicated in several mental health problems/disorders, including schizophrenia, mood disorders, anxiety, and suicidal behaviour [7]. ATP13A2 loss-of-function causes a disturbed intracellular polyamine distribution with (i) polyamine accumulation in late endolysosomes, resulting in rupture of these organelles and cathepsin B-mediated cell death [5], and (ii) a deficiency of cytosolic polyamines, leading to more mitochondrial derived reactive oxygen species causing oxidative stress [8]. Interestingly, all KRS variants characterized up until now show a (nearly) complete loss of ATP13A2 function as a consequence of protein mislocalization, instability, hampered autophosphorylation, dephosphorylation, and/or ATPase activity, or a combination thereof [3, 5, 9–13].

Although psychotic symptoms may occur in individuals with KRS (even in the absence of dopaminergic therapy), response to antipsychotic treatment has infrequently been described in the literature. As particular recommendations regarding the treatment of psychosis in this population remain scarce, we report a proband with KRS-associated psychosis caused by a homozygous novel loss-of-function *ATP13A2* variant, who ultimately responded well to quetiapine monotherapy. We also performed a review of the literature with respect to treatment response in individuals with psychosis.

## Case report

We report a 22 year old male with a mild intellectual disability (FSIQ = 73 in 2016) who presented to clinical attention after abruptly developing psychotic symptoms in 2019 at the age of 17. He was born around 36 weeks gestation via cesarean section due to either oligohydramnios or breech position (there have been conflicting reports in this respect). Although there were no gross motor delays he was described as being clumsier than his peers and later struggled with handwriting. He was delayed in both expressive and receptive speech and had articulation difficulties. Although he did not undergo a formal psychoeducational assessment until his teenage years (in 2016), he reportedly always struggled academically. Starting at around the time of his psychiatric admission in 2019 (but possibly earlier), his cognitive abilities began to progressively deteriorate. He nonetheless completed grade 12 thereafter, following his admission to hospital. Frank motor symptoms had not been identified or endorsed prior to the initiation of antipsychotic medication (see below for details). His medical history is otherwise only remarkable for intermittent low grade microcytic anemia and bilateral hydroceles (for which he underwent a bilateral hydrocelectomy in 2022).

**Fig. 1:**
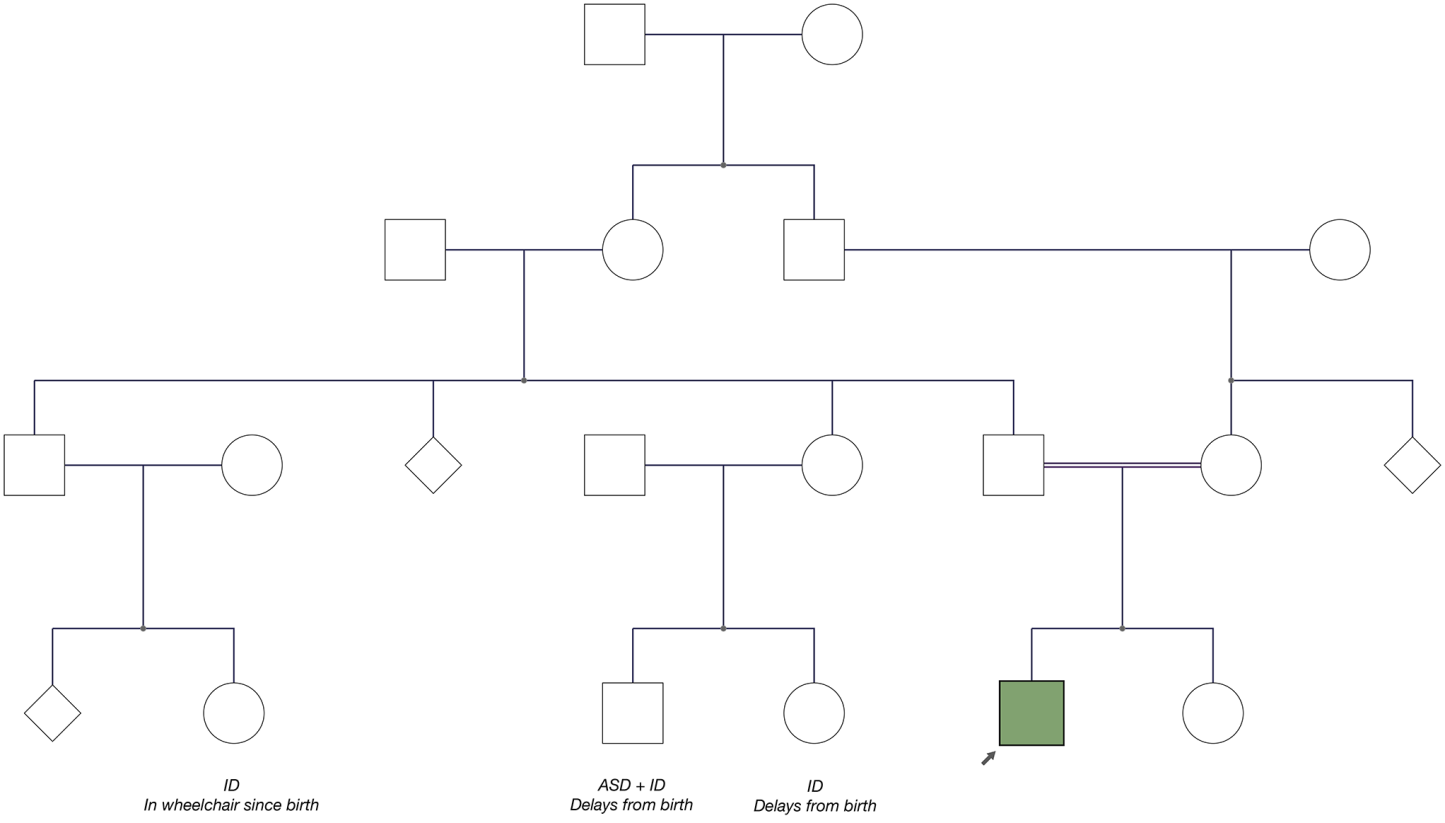
Pedigree

In terms of family history, he has a paternal first cousin who is intellectually disabled and non-ambulatory; however, her presentation is thought to be non-progressive. He has two additional paternal first cousins (who are siblings) who are autistic and/or intellectually disabled, and non verbal. There is otherwise no known history of parkinsonism, psychosis, or any other neuropsychiatric syndromes in any family members. A pedigree is provided in Fig. 1.

Apart from vaguely described anxiety, of a generalized and social nature, as well as possible attention-deficit/hyperactivity disorder, he was generally psychiatrically well prior to his index admission to hospital in 2019 (at age 17), which occurred immediately following a traumatic event. Although his symptoms were initially queried to represent a severe trauma reaction, the presence of frank psychotic symptoms eventually became evident, including disorganization of thought and behaviour, as well as persecutory, referential, and hyperreligious delusions that occurred in association with thematically congruent auditory hallucinations. For example, he became concerned that his father was “the devil”, and reported hearing “Satan” and “Jesus” speaking to him. In this context, he was also observed responding to internal stimuli. While his psychotic symptoms initially resolved with the initiation of low dose risperidone, he experienced remarkably severe extrapyramidal symptoms (EPS). As such, he was transitioned to olanzapine in the months following his discharge, which was comparatively less problematic from a motor side effect standpoint, but nonetheless continued to cause atypically severe EPS on doses as low as 2.5 mg qhs.

While a general medical workup was unrevealing, including a brain MRI which did not show obvious evidence of neuronal brain iron accumulation, his overall neuropsychiatric and developmental picture combined with a history of parental consanguinity, prompted a medical genetics service consult shortly before his discharge from hospital. While initial testing with chromosomal microarray was negative, clinical exome sequencing (Blueprint Genetics 2021) revealed a homozygous variant in *ATP13A2* (NM_022089.4, c.1970_1975del) which is predicted to cause an in-frame deletion of two amino acids, p.(Pro657_Glu658del). This variant is rare and absent from the gnomAD v4 database (accessed Nov 28, 2023). Although this in-frame deletion was initially formally classified as a variant of uncertain significance, it was clinically considered to be likely pathogenic given that his overall presentation was highly suggestive of KRS. Additionally, biochemical analysis demonstrating that the variant causes loss-of-function was performed (see below for details), confirming pathogenicity.

While his psychotic symptoms and comorbid anxiety were initially reasonably well controlled with low dose olanzapine following his discharge from hospital, he exhibited worsening dysarthria, dysphagia, bradykinesia, and balance difficulties over the subsequent years (between 2020 and 2022). He was assessed in a specialized movement disorders clinic in 2022 at age 20 and was found to exhibit new-onset marked difficulties with vertical eye movements in the upward plane with hypometric saccades in the horizontal plane. At the time of the assessment there was no blepharospasm and no orofacial dystonia. Marked hypomimia and hypophonia were noted. Moderate rigidity was noted axially as well as in both upper extremities with marked (slightly asymmetric) bradykinesia in all four limbs (more prominent in the left upper extremity and right lower extremity). He had no difficulty standing up from a seated position. Gait examination revealed markedly reduced stride length and arm swing, particularly in the left upper extremity. The pull test was positive. Deep tendon reflexes were brisk in the upper extremities at 3 + with no Hoffmann’s sign, and 2 + at the knees. No spasticity was appreciated. No tremor or any other abnormal movements were observed. On more recent examinations (beginning in early 2023), facial-faucial-finger mini-myoclonus has also been observed.

When attempts were made to lower his dose of olanzapine to 1.25 mg qhs, his motor symptoms predictably improved but he became more paranoid and anxious. As such, he was eventually cross-titrated from olanzapine to quetiapine in late 2022 at the age of 21. Although he experienced a reemergence of symptoms at certain points during the transition to quetiapine, he eventually again achieved remission of his psychotic symptoms on quetiapine monotherapy (initially on quetiapine XR 200 mg qhs in addition to quetiapine IR 100 mg qhs, before his dose of the XR formulation was increased to 300 mg due to a recurrence of occasional mild stress-induced paranoia). A combination of the extended release and immediate release formulations was chosen, as he experienced more frequent breakthrough symptoms in a diurnal pattern (specifically, in the evenings prior to his next dose) on the immediate release formulation alone. Additionally, as expected his motor symptoms noticeably improved with the change to quetiapine, despite some degree of ongoing dysarthria, rigidity, and bradykinesia, as well as continued reduced stride length and arm swing while walking.

## Methods

### Biochemical analysis of the ATP13A2 variant

*Cell culture* - Human neuroblastoma SH-SY5Y (RRID: CVCL_0019) cell lines either non-transduced (nts) or stably overexpressing wild-type ATP13A2 (Addgene plasmid #171485, RRID: Addgene_171485 or Addgene plasmid #213697, RRID: Addgene_213697), a catalytically dead variant (D508N) (Addgene plasmid #171820, RRID: Addgene_171820), or the Pro652_Glu653del ATP13A2 variant (Addgene plasmid #213700, RRID: Addgene_213700) (the nomenclature is based on the sequence of ATP13A2 splice variant 2, which is 5 amino acids shorter at the N-terminal region and has been historically used for biochemical analysis [5]) were generated via lentiviral transduction as described previously [14]. A detailed protocol can be found at 10.17504/protocols.io.bw57pg9n. Cells were maintained at 37 °C in the presence of 5% CO2 and incubated in high-glucose Dulbecco’s modified Eagle medium supplemented with 1% penicillin/streptomycin (Sigma), 15% fetal calf serum (heat inactivated) (Sigma), 1% non-essential amino acids (Sigma), 1% sodium pyruvate (Gibco), and selection antibiotic (160 µg/mL hygromycin or 2 µg/mL puromycin (Invivogen)). The treatments were performed in the same medium, with the exclusion of selection antibiotic.

*Immunofluorescence* - Cells were seeded at 75 000 cells/well in a 12-well plate with coverslips and allowed to attach and grow for 48 h. Thereafter, cells were washed with ice-cold PBS and fixed with 4% paraformaldehyde (Thermo Fisher Scientific) (30 min, 37 °C). Cells were washed twice more with ice-cold PBS before permeabilization and blocking with a mixture of 5% BSA (Roth) and 0.5% saponin (Sigma) (referred to as blocking buffer) for 1 h. Next, cells were incubated with primary antibody (anti-ATP13A2, A3361, Sigma, RRID: AB_10597403; anti-CD63, 11-343-C100, ExBio, RRID: AB_10733918) (diluted 1/200 in blocking buffer) for 2 h and then subjected to secondary antibody (Alexa-488 goat anti-rabbit IgG, R37116, Invitrogen, RRID: AB_2556544; Alexa-594 goat anti-mouse IgG, A11005, Invitrogen, RRID: AB_2534073) (diluted 1/1000 in blocking buffer) for 30 min on a shaker. Subsequently, cells were immersed in 200 ng/ml DAPI (D9542, Sigma) for 15 min. Samples were thoroughly washed with PBS in between the different steps. Finally, the samples were mounted and images were acquired using a LSM880 microscope (Zeiss) with a 63x objective and Airyscan detector. A detailed protocol can be found at: 10.17504/protocols.io.bp2l6xy3klqe/v1.

*SDS-page and immunoblotting* – 70% confluent cells were harvested and subsequently lysed with radio-immunoprecipitation assay buffer (Thermo Fisher Scientific) supplemented with protease inhibitors (Sigma). 10 µg of protein (concentration determined with a bicinchoninic acid protein assay) was then loaded on a NuPage 4–12% Bis-Tris gel (Thermo Fisher Scientific, Bio-Rad) and subjected to an electrophoresis run at 130 V in MES running buffer (Thermo Fisher Scientific). The proteins were subsequently transferred to a PVDF membrane in NuPage transfer buffer (Thermo Fisher Scientific) supplemented with 10% v/v methanol (Roth). Immunoblots were blocked by incubation in 5% w/v milk powder (1 h, room temperature). Next, immunoblots were probed with primary antibodies (anti-GAPDH, G8795, Sigma, RRID: AB_1078991; anti-ATP13A2, A3361, Sigma, RRID: AB_10597403) (diluted 1/5000 and 1/1000 in 1% w/v BSA, respectively) and incubated O/N (4 °C). After thorough washing, immunoblots were subjected to secondary antibodies (HRP-linked anti-mouse IgG, 7076 S, Cell Signaling, RRID: AB_330924; HRP-linked anti-rabbit IgG, 7074 S, Cell Signaling, RRID: AB_2099233) (diluted 1/2000 in 1% w/v milk powder) (1 h, room temperature). All dilutions and wash steps were performed with TBS supplemented with 0.1% v/v Tween-20 (PanReac AppliChem). Detection was performed by means of chemiluminescence (Bio-Rad ChemiDoc) and protein levels were quantified with Image Lab software (RRID: SCR_014210, version 6.0.1, Bio-Rad, https://www.bio-rad.com/en-be/product/image-lab-software?ID=KRE6P5E8Z). A detailed protocol can be found at: 10.17504/protocols.io.e6nvwdqz2lmk/v1.

^*14*^*C-labeled polyamine uptake* – This protocol is based on a previous publication [15], with minor modifications. Briefly, 70% confluent SH-SY5Y cells in a 12-well plate were incubated (30 min, 37 °C) either with 5 µM ^14^C-labeled spermine (3139-50 µCi, ARC) or with a mixture of 5 µM ^14^C-spermine and 100 µM unlabeled spermine in cell culture medium. The medium was subsequently aspirated and cells were washed twice with ice-cold PBS (without Ca^2+^ and Mg^2+^). Next, the cells were lysed by incubation in 200 µl radio-immunoprecipitation assay buffer (10 min, room temperature) (Thermo Fisher Scientific) before scraping. The cell lysate was added to scintillation vials filled with 7 ml EcoLite Liquid Scintillation Cocktail (01882475-CF, MP Biomedicals). Thereafter, the wells were washed with 200 µl ice-cold PBS (without Ca^2+^ and Mg^2+^), which was added to the accompanying scintillation vial. Finally, ^14^C radioactivity in counts-per-minute (CPM) was measured with liquid scintillation counting (TRI-CARB 4910TR V Liquid Scintillation Counter, PerkinElmer). A detailed protocol can be found at: 10.17504/protocols.io.j8nlkorm5v5r/v1.

*Microsome collection -* SH-SY5Y cells overexpressing wild-type or mutant ATP13A2 were seeded in 500 cm^2^ plates. Once they reached 70–80% confluency, cells were collected. Next, cells were lysed by resuspending the cell pellet in 3 ml hypotonic LIS buffer (10 mM Tris.HCl pH 7.5, 0.5 mM MgCl_2_.6H_2_O, 1 mM DTT, 1x SigmaFast protease inhibitors) (S8830, Merck), which was incubated on ice for 15 min. The suspension was transferred to a Dounce homogenizer and 60 up-and-down strokes were applied, followed by addition of 3 ml 1 M solution (0.5 M sucrose, 10 mM Tris.HCl pH 7.3, 40 µM CaCl_2_, 1 mM DTT, 1x SigmaFast protease inhibitors) and another 30 up-and-down strokes. The nuclear (1000 x g, 10 min, 4 °C), mitochondrial-lysosomal (12 000 x g, 20 min, 4 °C), and microsomal fractions (140,000 x g, 35 min, 4 °C) were then collected. Fractions were suspended in 0.25 M sucrose with 1x SigmaFast protease inhibitors. A detailed protocol can be found at: 10.17504/protocols.io.5qpvo3w7dv4o/v1.

*ADP-Glo assay -* ATPase activity was assessed using a commercially available luminescence assay (ADP-Glo Max assay, V7002, Promega) according to manufacturer’s instructions. A reaction mixture (final volume 25 µl) was made in a 96-well plate and contained 50 mM MOPS-KOH (pH 7), 100 mM KCl, 11 mM MgCl_2_, 1 mM DTT, 0.1 mg/ml DDM, 5 µg microsomes (1:2 ratio DDM: microsomes) and various concentrations of polyamines. The microsomes were collected from SH-SY5Y cells overexpressing ATP13A2 (wild-type or mutants). Next, the reaction was incubated (20 min, 37 °C followed by 10 min, room temperature) following the addition of 5 mM ATP and terminated by adding 25 µl of ADP-Glo Reagent. The 96-well plate was subsequently incubated (40 min, room temperature), followed by the addition of 50 µl of ADP-Glo Detection Reagent. After 60 min, luminescence was detected using the Bio Tek plate reader. A detailed protocol can be found at: 10.17504/protocols.io.3byl4q1x8vo5/v1.

*RNA collection and qPCR -* RNA was isolated using the NucleoSpin RNA plus kit (Macherey-Nagel) following manufacturer’s instructions. RNA concentration and purity were determined using a Nanodrop spectrometer (Thermo Fisher Scientific). 5 µg RNA was then converted to cDNA using the High-Capacity cDNA Reverse Transcription Kit (Thermo Fisher Scientific). A 96-well plate was prepared with a ten-fold dilution of each cDNA sample in duplicates (with a volume of 5 µl cDNA per well), to which the following reaction mixture was added (volumes are given per well): 10 µl SYBR Green master mix (Roche), 1 µl of 5 µM forward primer, 1 µl of 5 µM reverse primer, and 3 µl distilled water. The cDNA was replaced by an equivalent volume of distilled water for the negative controls. Primers targeting the *ATP13A2* gene were 5’-CATGGCTCTGTACAGCCTGA-3’ (forward) and 5’ CTCATGAGCACTGCCACTGT-3’ (reverse). Primers targeting the *β-actin* gene were 5’-CACTGAGCGAGGCTACAGCTT-3’ (forward) and 5’-TTGATGTCGCGCACGATTT-3’ (reverse). The qPCR read-out was performed, in which the reaction was initiated at 95 °C for 10 min, followed by 50 cycles at 95 °C for 10 s and 55 °C for 30 s, and ended at 95 °C for 1 min. A melting curve was determined from 55 to 95 °C. A detailed protocol can be found at: 10.17504/protocols.io.14egn3ymzl5d/v1.

### Literature search

**Fig. 2:**
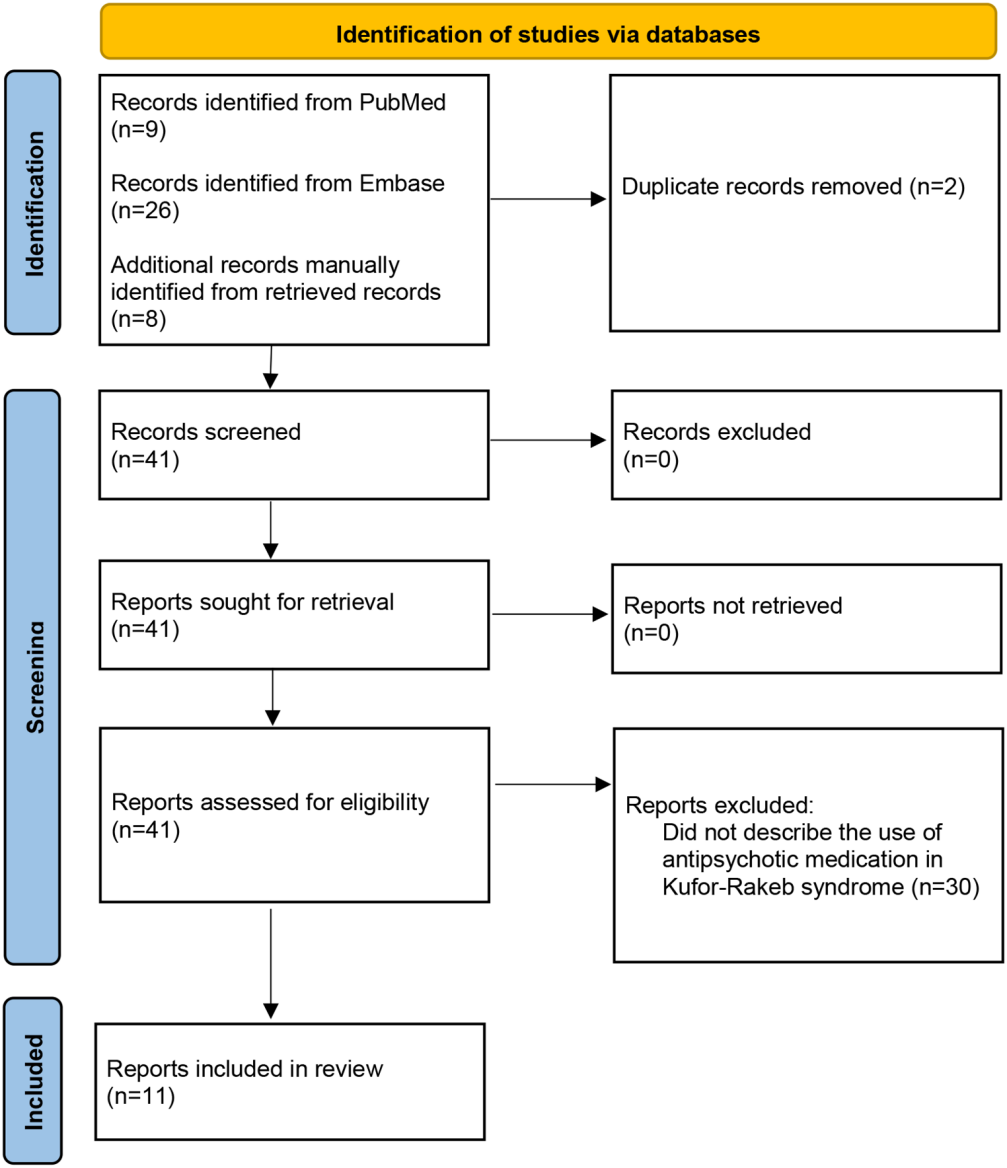
Literature Search flow diagram for antipsychotic use in Kufor-Rakeb syndrome

PubMed and Embase searches were completed in April 2023 (and updated in December 2023) using the terms “kufor-rakeb”, “kufor rakeb”, “KRPPD”, ”parkinson disease-9”, “parkinson disease 9”, “PARK9”, “pallido-pyramidal degeneration with supranuclear upgaze paresis and dementia”, “ATP13A2”, or “1p36.13”, in combination with “schizophrenia”, “psychosis”, “psychotic”, “hallucinations”, “delusions”, “paranoia”, “antipsychotic”, or “neuroleptic”. The Pubmed search yielded nine results and the Embase search yielded 26. After duplicates were removed, 33 results remained. All results were manually reviewed, including their reference lists, for additional relevant articles. OMIM was also reviewed. Only articles that described the use of antipsychotic medications in individuals with Kufor-Rakeb syndrome were included in the review. The search process is outlined in Fig. 2.

## Results

### Biochemical analysis of the ATP13A2 variant

**Figure 3:**
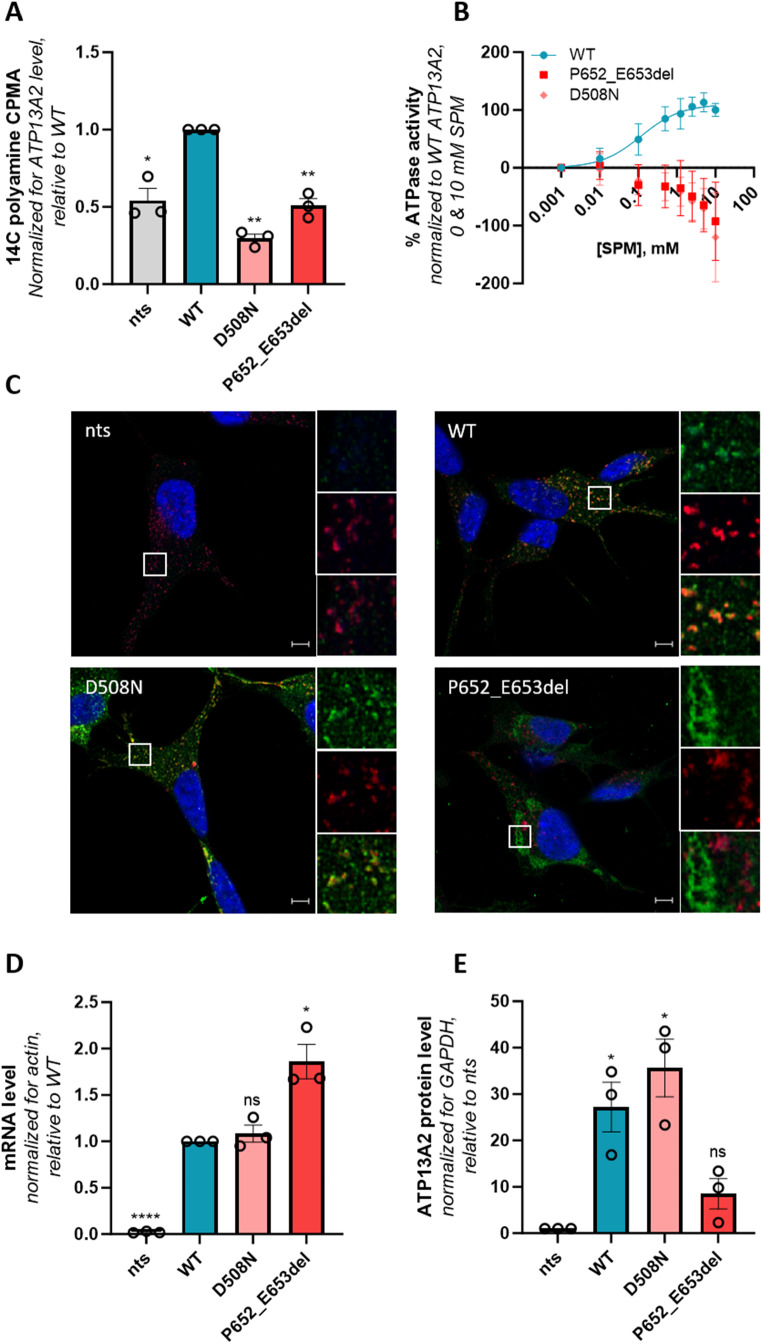
Functional characterization of the Pro652_Glu653del ATP13A2 variant. SH-SY5Y neuroblastoma cells either non-transduced (nts) or stably transduced with constructs encoding for wild-type ATP13A2 (WT), a catalytically dead variant (D508N), or the Pro652_Glu653del (P652_E653del) ATP13A2 variant were used to analyze (**A**) cellular polyamine uptake potential by scintillation counting following a 30 min incubation with ^14^C-labeled spermine. CPMA, counts-per-minute (*n* = 3). (**B**) Spermine-induced ATPase activity was determined in isolated microsomes (*n* = 3). Fitting was performed using the non-linear allosteric sigmoidal. (**C**) Colocalization of ATP13A2 (in green) with the late endolysosomal marker CD63 (in red) was analyzed by immunofluorescence (*n* = 3). Scale bar, 5 μm. Individual and merged channels are shown for the boxed areas. ATP13A2 mRNA (*n* = 3) (**D**) and protein (*n* = 3) (**E**) levels in the SH-SY5Y cells were assessed via qPCR and immunoblotting and qPCR, respectively. *, *p* < 0.05; **, *p* < 0.01; ****, *p* < 0.0001; ns, non-significant *versus* WT (**A, D**) or nts (**E**); one-sample t-test. Graphs were created with GraphPad Prism (RRID: SCR_002798, Prism - GraphPad)

To functionally characterize the novel *ATP13A2* variant described in this study, we generated SH-SY5Y neuroblastoma cells overexpressing either wild-type ATP13A2, a catalytically dead variant that is defective in auto-phosphorylation (D508N, functioning as a negative control) or the Pro652_Glu653del variant. Cells overexpressing Pro652_Glu653del presented a significantly lower uptake of ^14^C-labeled spermine as compared to cells overexpressing wild-type ATP13A2 (Fig. 3A), which offers a read-out for ATP13A2’s transport activity. This correlated well with the spermine-dependent ATPase activity in the membrane fractions of these cell lines. The ATPase activity of the Pro652_Glu653del protein was comparable to the D508N loss-of-function mutant pointing to complete loss of activity (Fig. 3B). Whereas wild-type and D508N ATP13A2 colocalized with CD63, a late endolysosomal marker, the Pro652_Glu653del variant displayed a mesh-like pattern suggesting mislocalization in the endoplasmic reticulum (Fig. 3C). We also analyzed the expression level, and found that Pro652_Glu653del mRNA levels were significantly higher than wild-type (Fig. 3D), demonstrating successful transduction. In contrast, the Pro652_Glu653del variant exhibited a lower protein expression than wild-type or D508N ATP13A2 (Fig. 3E), suggesting that protein stability may be impaired. In conclusion, in line with other previously described KRS variants [5], the Pro652_Glu653del variant exhibits full loss-of-function, which can be explained by a combination of protein instability, mislocalization, and inactivity. Our biochemical analysis indicates that in a homozygous context, this variant may be disease causing.

### Literature search

Although psychotic symptoms have often been described in KRS, the effectiveness of antipsychotic therapy has only been described for six individuals [16–22]; specifically with respect to olanzapine [20], aripiprazole [21, 22], quetiapine [16, 20], clozapine [19], and thioridazine (which unsurprisingly led to a worsening of motor symptoms) [18]. Although at least a partial and/or temporarily sustained therapeutic response was reported in all cases, it is not clear that any individuals achieved a full and enduring remission of psychotic symptoms. The one possible exception is the individual described by Balint et al. [21] who eventually “remained off antipsychotics without paranoid outbreaks”; however, no further details were provided, and there is also no mention of whether her visual hallucinations recurred. Additional details pertaining to the individuals’ psychiatric phenotypes and response to treatment, as well as to their particular *ATP13A2* variants, are outlined in Table 1. Developmental and neurological history information is presented in Table 2. Additionally, narrative summaries for each case are provided in Supplemental Table 1 (outlining their psychiatric phenotypes) and Supplemental Table 2 (outlining their developmental and neurological phenotypes). Five additional articles alluded to the use of antipsychotic medication but did not provide any information regarding treatment response [23–27]. The clinical details pertaining to the psychiatric aspects of these additional five cases are outlined in Supplemental Table 3.

**Table 1:** Psychotic symptoms and response to treatment in previous cases

Study	Variant	Psychotic symptoms	Dopaminergic therapy*	Antipsychotic medications (total daily dose)	Treatment response	Antipsychotic impact on motor functioning
Di Fonzo et al. [16]; Chien et al. [17]	Homozygous missense variant in *ATP13A2* NM_022089.1, c.[1510 g > c]; c.[1510 g > c], p.[(Gly504Arg)]; [(Gly504Arg)]	VH	yes	1. haloperidol (NR) 2. quetiapine (50 mg)	1. NR 2. temporary improvement	1. NR 2. unclear
Behrens et al. [18]	Compound heterozygous variants in *ATP13A2*, no transcript reported: c.[3057delC]; [c.1306 + 5G > A]	AH, VH, paranoia, confusion	NR	thioridazine (25 mg)	improvement	worsening
Abbas et al. [19]	Homozygous missense variant in *ATP13A2*, reported on Ensembl transcript ENST00000341676: c.[2525T > C]; [2525T > C], p.[(Leu842Pro)]; [(Leu842Pro)]	AH, VH, “excessive fear”	yes	1. risperidone (NR) 2. olanzapine (NR) 3. quetiapine (NR) 4. levosulpiride (NR) 5. clozapine (25 mg)	1. NR 2. NR 3. NR 4. NR 5. improvement	1. NR 2. NR 3. NR 4. NR 5. NR
Pietrzak et al. [20]	Compound heterozygous variants in *ATP13A2*, no transcript reported: c.[2366_2367delTC]; [2209 C > T], p.[(Leu789Argfs*15)]; [(Gln737*)]	AH, paranoia	yes	1. olanzapine (2.5 mg) 2. quetiapine (50 mg)	1. temporary improvement 2. temporary improvement (in combination with olanzapine)	1. NR 2. NR
Balint et al. [21]	Compound heterozygous variants in *ATP13A2* NM_022089.4: c.[1472_1473del]; [c.2567_2568del], p.[(Gln491Argfs*29)]; [(Pro856Argfs*26)]	VH, paranoia	yes	1. “neuroleptics” (NR) 2. aripiprazole (2.5 mg)	1. NR 2. improvement, possibly remission	1. worsening 2. worsening
McNiel-Gauthier et al. [22]	Homozygous variant in *ATP13A2,* no transcript reported: c.[2126G > C]; [2126G > C], p.[(Arg709Thr)]; [(Arg709Thr)]	auditory and visual illusions, paranoia, ideas of reference	yes	aripiprazole (2–3 mg)	improvement	no definitive worsening

AH = auditory hallucinations; NR = not reported; VH = visual hallucinations

*variability existed across cases regarding when dopaminergic therapy was started relative to psychosis onset

**Table 2:** Developmental and neurological phenotypes of previous cases

Study	Developmental delay	Motor symptom age of onset (years)	Vertical gaze palsy	Facial-faucial-finger minimyoclonus	Spasticity	Cognitive impairment/deterioration	Neuroimaging findings
Di Fonzo et al. [16]; Chien et al. [17]	none	12	yes	NR	yes	none	diffuse moderate cerebral and cerebellar atrophy (CT)
Behrens et al. [18]	NR	18	yes	yes	yes	unclear; only “bradypsychia” was reported	diffuse atrophy (CT)
Abbas et al. [19]	NR	21	yes	NR	yes	yes	diffuse cerebral atrophy (CT); diffuse cerebral and brainstem atrophy (MRI)
Pietrzak et al. [20]	yes	17	yes	yes	yes	yes	cerebellar, brainstem, and mild cerebral atrophy (MRI)
Balint et al. [21]	none	27	yes	NR	yes	yes	cerebellar, parietal, and corpus callosal atrophy, in addition to “mild hyperintensity of the forceps minor of the corpus callosum”, “with a subtle ‘ear of the lynx’ sign” noted on the left (MRI)
McNiel-Gauthier et al. [22]	yes	26	yes	NR	yes	yes	diffuse atrophy and corpus callosal hypoplasia (MRI)

NR = not reported

## Discussion

Interestingly, various antipsychotic medications known to cause EPS were used in most previous case reports, despite KRS being a form of early-onset parkinsonism. In some cases, even typical or first generation antipsychotics, which are particularly problematic in this respect, were used. Although McNiel-Gauthier et al. [22] suggested that low dose aripiprazole was effective without definitive evidence of medication-related EPS in their patient, this was presumably speculative given his progressive motor dysfunction.

Quetiapine and clozapine, which are commonly used in PD when antipsychotic therapy is deemed necessary, were only used in a few cases. Specifically, quetiapine was only used in three patients [16, 19, 20], and its effectiveness and/or impact on motor functioning were only vaguely described by Di Fonzo et al. [16] and Pietrzak et al. [20]. Moreover, the concurrent use of levodopa in both cases [16, 20], as well as the use of olanzapine in one [20], further confounds interpretation of quetiapine’s effectiveness and tolerability in these patients. Clozapine (at a very low dose) has only been used in one published case to date [19]. Although this patient’s hallucinations reportedly improved, it is not clear that their symptoms fully remitted.

Here, we describe a KRS patient carrying a novel homozygous variant in *ATP13A2*, which was demonstrated to cause a complete loss of protein activity, in accordance with previously characterized *ATP13A2* KRS-causing variants [3, 5, 9–13]. Importantly, this is the first report of a patient with KRS whose psychotic symptoms remitted with quetiapine monotherapy. Notably however, Di Fonzo et al. [16] and Pietrzak et al. [20] both utilized a total daily dose of 50 mg, whereas we ultimately had to target a higher dose (400 mg total daily dose). Although quetiapine has so far been well tolerated in our patient, we cannot be certain that it has not contributed to his ongoing motor symptoms, as olanzapine and quetiapine were cross-titrated. That is, at no point was he off of antipsychotic medication entirely; however, he and his family observed significant improvement with respect to his motor functioning following the switch to quetiapine. It should also be noted that at the time of publication, our patient was not yet on dopaminergic therapy of any kind, and it is possible that quetiapine may prove less effective should concurrent levodopa treatment eventually be required. Similarly, quetiapine may prove to be less effective and/or well tolerated as his illness progresses.

Although controlled trials in neurodegenerative parkinsonian disorders have been disappointing [28], quetiapine is nonetheless commonly prescribed for PD psychosis [29] and similarly represents a reasonable option in patients with KRS, given its side effect profile and ease of use. Although clozapine has more evidence in the treatment of PD psychosis [30], we did not pursue a clozapine trial given that our patient’s psychotic symptoms remitted with quetiapine therapy. Although clozapine can also be considered, particularly when psychotic symptoms are treatment resistant and provided no medical contraindications exist, its use is limited by the possibility of rare but potentially serious side effects and the related need for regular blood monitoring. It is also worth noting that no previous articles have described the use of pimavanserin in KRS-associated psychosis, despite pimavanserin being the only FDA approved medication for the treatment of PD psychosis in the United States [30]. This was not an option for our patient, as it is currently not available in our region.

## Conclusion

This report characterizes a homozygous novel loss-of-function *ATP13A2* variant in an individual with KRS. This is also the first description of psychotic symptoms remitting in response to quetiapine monotherapy in KRS. Given its lower propensity to cause EPS, quetiapine should be considered in the management of KRS-associated psychosis when antipsychotic therapy is deemed necessary.

## Electronic supplementary material

Below is the link to the electronic supplementary material.

Supplementary Material 1

Supplementary Material 2

Supplementary Material 3

## Data Availability

All datasets generated or analyzed in this study can be found through the Zenodo repository (doi:10.5281/zenodo.10600748). All experimental protocols are shared via protocols.io. For the purpose of open access, the author has applied a CC BY 4.0 public copyright license to all Author Accepted Manuscripts arising from this submission.

## Declarations

### Submission declaration

A longer version of this manuscript has previously been posted on the preprint server medRxiv (https://www.medrxiv.org/content/10.1101/2024.01.12.23300401v2). It has otherwise not been published elsewhere and is not under consideration for publication elsewhere.
