# Supplementary material for: Kufor-Rakeb syndrome-associated psychosis: a novel loss-of-function ATP13A2 variant and response to antipsychotic therapy
Source: Neurogenetics. 2024 Jul 18;25(4):405–15. doi: 10.1007/s10048-024-00767-7 (PMC11534834; doi:10.1007/s10048-024-00767-7)
Supplement: Supplementary file 1 — Supplementary Material 1 [file 10048_2024_767_MOESM1_ESM.docx]

**Supplemental Table 1. Narrative Summaries of Psychiatric Phenotypes and Response to Treatment in Previous Cases**

| **Study** | **Psychiatric Phenotype and Treatment Response** |
| --- | --- |
| Di Fonzo et al. [16]; Chien et al. [17] | Di Fonzo et al. [16] were the first to describe the treatment of psychotic symptoms in a patient with KRS; a male who developed visual hallucinations, and eventually aggression, as a young adult after the initiation of levodopa and bromocriptine. While he was admitted to hospital and treated with haloperidol, the effects it had on his psychotic and motor symptoms were not described. Nonetheless, it is stated that bromocriptine was stopped and a combination of levodopa/benserazide and quetiapine “allowed satisfactory control” of his motor and psychotic symptoms. However, during the year prior to his assessment by the authors of the report, his parkinsonism worsened “with re-emergence of hallucinations and severe fluctuations and dyskinesias”, requiring changes to his medication regimen. At the time of the assessment he continued to experience “psychiatric disturbances” during “on” states and his medications included levodopa (plus benserazide) 350 mg/day and quetiapine 50 mg/day. Following his death, a subsequent paper by Chien et al. [17] described this individual’s neuropathological characteristics, but no additional information regarding his psychiatric phenotype or response to treatment was provided. |
| Behrens et al. [18] | The authors described numerous members of an affected family, including one who developed episodes of confusion and “paranoid auditory hallucinations” in adulthood, seemingly in response to trihexylphenidate therapy. The occurrence of visual hallucinations is also mentioned in a table but not described in detail. Thioridazine 25 mg/day “improved” his hallucinations but worsened his EPS. While two siblings also experienced hallucinations, it is not reported if they were treated with antipsychotic medication. |
| Abbas et al. [19] | The authors described a 32 year old male who developed “excessive fear” as well as auditory and visual hallucinations at age 27, in addition to hypersexuality at age 29, in the context of longstanding ropinirole therapy. While it is noted that he had been treated with risperidone, olanzapine, quetiapine, and levosulpiride, his response to these medications is not described. At 32 years of age the initiation of clozapine 25 mg/day (in addition to clonazepam 2 mg/day) led to a reduction in his hallucinations, despite ongoing treatment with levodopa/carbidopa 200/50 mg tid (ropinirole had been stopped). |
| Pietrzak et al. [20] | The authors described a 28 year old female who at age 25 developed psychosis characterized by “persecutory delusions of a poorly defined imminent threat and auditory hallucinations (scary voices)”. Her symptoms were reportedly “controlled with olanzapine”. At the time of the initial assessment by the authors, her medication regimen included levodopa with benserazide “50 mg + 12.5 mg tid”, quetiapine 25 mg bid, and olanzapine 2.5 mg/day. While her levodopa was increased to “100 mg + 25 mg qid”, “an attempt at further increase” resulted in hypotension and a return of persecutory delusions. Paroxetine was also prescribed for anxiety. |
| Balint et al. [21] | The authors described a 38 year old female who developed paranoia at age 24, characterized by delusions of infidelity involving her husband as well as a belief that her work colleagues were “going through her notes” and talking “behind her back”. She was also diagnosed with bulimia nervosa at some point in adulthood. Her symptoms worsened at age 27, such that she “developed psychosis with complex persecutory delusions with episodes of visual hallucinations”. Notably, she was not receiving dopaminergic therapy at this time, as she had not yet been diagnosed with KRS. “Antidepressants and neuroleptics” caused “sleepiness, slowness, and clumsiness”, even at low doses. Although her “psychiatric symptoms” waxed and waned while her medication was adjusted, her response to treatment during this period is not described in detail. While aripiprazole eventually “stabilized her”, it is unclear if her psychotic symptoms fully remitted. Although her maximum dose of aripiprazole is not specified, at age 32 she was taking 2.5 mg/day. Aripiprazole was stopped shortly thereafter (following the diagnosis of KRS) which led to a mild improvement in her “slowness”. However, it is not reported if this change also led to an improvement in her other motor symptoms or a worsening of her psychotic symptoms. However, the authors do state that the initiation of levodopa/carbidopa 300 mg/day led to an improvement in her tremor and bradykinesia, and that from age 32 onward she was able to remain off antipsychotic medication “without paranoid outbreaks”. |
| McNiel-Gauthier et al. [22] | The authors described a 32 year old male who was referred to psychiatric services due to “behavioral outbursts” and “odd beliefs”. In adulthood, he developed “ideas of reference” (e.g., in relation to the television) and “non-systematized persecutory delusions” that involved concerns about being “mocked” by others and surveyed from parked cars. Relatedly, he experienced visual and auditory illusions but no frank hallucinations. Such symptoms were described as occurring daily and episodically, and were reported to be worse after watching television and using the computer. He was also oppositional and aggressive in this context. Lorazepam was initially “mildly successful” in helping “control these outbursts”, but led to sedation. Aripiprazole 2 mg/day was trialed and after three weeks he exhibited a “moderately reduced frequency of ideas of reference” and became “less preoccupied” with his persecutory delusions. While his global improvement score was “2” (“much improved”), he still rarely exhibited “impulsive and disinhibited behaviors”. With respect to EPS, only his dysarthria seemingly worsened. His psychotic symptoms and EPS remained unchanged after three additional months of being on aripiprazole 2 mg/day. While he was weaker, this was attributed to the natural course of the disease and not to the use of aripiprazole. After one year of treatment he experienced a recurrence of “behavioral outbursts” three to four times/week, which was also thought to be related to disease progression. As a result, his dose of aripiprazole was increased to 3 mg/day with a “good response”. Approximately two years after the initial assessment, despite continued neurological deterioration his psychotic and behavioural symptoms remained “stable”. Although periodic “behavioural outbursts” persisted, albeit less frequently, his psychotic symptoms in particular were “much improved” compared to the first assessment. Overall, the authors concluded that aripiprazole therapy resulted in “few residual psychotic symptoms” “without definite medication related motor side effects”. |
