# Supplementary material for: Kufor-Rakeb syndrome-associated psychosis: a novel loss-of-function ATP13A2 variant and response to antipsychotic therapy
Source: Neurogenetics. 2024 Jul 18;25(4):405–15. doi: 10.1007/s10048-024-00767-7 (PMC11534834; doi:10.1007/s10048-024-00767-7)
Supplement: Supplementary file 2 — Supplementary Material 2 [file 10048_2024_767_MOESM2_ESM.docx]

**Supplemental Table 2. Narrative Summaries of the Developmental and Neurological Phenotypes of Previous Cases**

| Study | Developmental and Neurological History |
| --- | --- |
| Di Fonzo et al. [16]; Chien et al. [17] | The individual’s development was normal. At age 12 he was noted to be physically slow with “awkward” and “clumsy” movements and was diagnosed with juvenile Parkinson’s disease. His motor symptoms initially responded to levodopa and bromocriptine, but he soon thereafter developed choreic dyskinesias in addition to visual hallucinations. Bromocriptine was stopped and entacapone was eventually trialed. He was later described as having severe akinetic-rigid parkinsonism with limited upward gaze and spasticity. Bradykinesia and postural instability were also noted. A very mild tremor was observed in the lower lip and chin when speaking, but he never exhibited a tremor in the extremities. |
| Behrens et al. [18] | The individual “hardly finished 5th grade at age 13” and exhibited “slowness and rigidity” five years later. Notable features on examination at age 24 included “bradypsychia”, “monotonous voice", stooped gait, reduced arm swing, left arm and bilateral wrist cogwheel-rigidity, and ankle clonus. Trihexylphenidate “had a mild effect”. At age 38 he was described as being “apathetic, with an unstable, stooped gait, upward gaze palsy, and facial-faucial-finger minimyoclonus”. Bradykinesia, hypomimia, spasticity, paresis, and positive palmomental reflex were also reported. He was wheelchair-bound by age 32. |
| Abbas et al. [19] | At 21 years of age the individual began to experience an insidious onset of “progressive slowness in activities of daily living, change in speech, forgetfulness, and falls”. His slowness improved with levodopa therapy but he developed dyskinesias after three years, so levodopa was stopped in favour of ropinirole and trihexyphenidyl. His gait, speech, and cognition worsened over the following 10 years and at some point he experienced urinary and bowel incontinence. 10 years following the onset of symptoms his Mini-Mental State Examination score was 8/30. Upward gaze was impaired and there was an absence of horizontal and vertical saccades. Spastic dysarthria was also noted, as well as torticollis to the left and “jaw-opening dystonia while performing rapid alternate hand movements”. Additional pertinent examination features included generalized spasticity, truncal myoclonus, a resting tremor in both upper extremities (with intention and postural components) as well as myoclonic jerks, bradykinesia, and rigidity. Ropinirole and trihexyphenidyl were stopped and he was treated with levodopa thereafter leading to a “mild improvement in slowness”. However, he continued to experience truncal myoclonus and a severe intention tremor. |
| Pietrzak et al. [20] | The individual “ceased to sit unsupported and started to cross her lower limbs  when held upright” at six months of age. At 11 months examination revealed calcaneal tendon contractures and spastic tetraplegia. She was able to stand on her own by 13 months of age and walked at 20 months. At the same time her language skills were “quickly developing” and she was able to speak in simple sentences. She initially “performed well” in school but ran slower than her peers. Learning difficulties were not apparent until grade 10, when she particularly began to struggle with math; she transitioned to vocational school after one year. She experienced falls at age 17 and eventually began to exhibit a shuffling gait. At age 23 examination revealed a tongue tremor, dysarthria, “monotonous speech”, “limb dysmetria and dysdiadochokinesis”, a positive Tromner sign, and a wide-based gait. She was bedridden by age 25 and had additionally developed dysphagia and excessive drooling. At that time examination revealed impaired upward gaze, severe bradykinesia, and quadrupedal spasticity. Severe hypokinetic dysarthria, hypomimia with a “dystonic smile”, occasional blepharoclonus, and perioral myoclonias were also noted, in addition to absent upward saccades and hypometric/delayed downward/horizontal saccades. Additional findings included lead pipe rigidity and spasticity, frontal release signs, Jacobsohn, Sterling, and Rossolimo signs, facial-faucial-finger minimyoclonus, and upper extremity ataxia. Levodopa led to an improvement in rigidity, speech, and swallowing. |
| Balint et al. [21] | Although developmental milestones were normal, this individual was described as being “clumsier and slower” than peers as a child. Her school performance was “average”. Although her psychiatric symptoms developed years prior, motor symptoms (i.e., “slowness” and “clumsiness”) were not identified until age 27 in the context of low dose aripiprazole therapy. While her “clumsy gait” and fine motor skill problems persisted on aripiprazole, her neurological phenotype was not well characterized until age 32 following a pregnancy. At that time hypomimia, frontalis overactivity, and scanning dysarthria were noted. There was upward gaze restriction and she had a tendency to “blink and use a head thrust when initiating saccades”. Left sided palmomental reflex, “global slowness”, mild bradykinesia, a “mild irregular postural tremor”, ankle clonus, leg spasticity, and a “wide-based spastic gait” without arm swing were also observed. Neuropsychological testing revealed multi-domain cognitive impairment. Thereafter she experienced progressive cognitive impairment and developed limb dysmetria, distal lower limb weakness, and a resting left hand tremor. Levodopa resulted in clinical improvement. |
| McNiel-Gauthier et al. [22] | The individual experienced developmental delay and learning difficulties, and was described as having “limited autonomy in instrumental activities of daily living”. Nonetheless, he apparently graduated from high school with supports. He developed progressive spastic quadriparesis and his first “major reported spastic episode” occurred at age 26. Ataxia, “pyramidal tract dysfunction”, vertical gaze palsy, and cognitive impairment were also noted but no additional information was provided. Although further timeline details regarding his neurological symptoms were not reported, it is noted that his spasticity and dysarthria worsened through early adulthood. Progressive motor weakness and a leg tremor were later described. His response to levodopa was “limited”. He was also diagnosed with epilepsy at age 24. |
