# Supplementary material for: Kufor-Rakeb syndrome-associated psychosis: a novel loss-of-function ATP13A2 variant and response to antipsychotic therapy
Source: Neurogenetics. 2024 Jul 18;25(4):405–15. doi: 10.1007/s10048-024-00767-7 (PMC11534834; doi:10.1007/s10048-024-00767-7)
Supplement: Supplementary file 3 — Supplementary Material 3 [file 10048_2024_767_MOESM3_ESM.docx]

**Supplemental Table 3. Additional Articles that Reference Antipsychotic Use**

| Study | Clinical Information |
| --- | --- |
| Williams et al. [23] | Four affected family members were diagnosed clinically with Kufor-Rakeb syndrome, but at the time of publication had not undergone confirmatory genetic testing. All four individuals experienced visual hallucinations. However, only one of whom is reported to have received antipsychotic medication. This individual was a 36 year old male who was treated with risperidone 1 mg/day in addition to levodopa/carbidopa 250 mg bid and biperiden 2 mg/day. It is not stated if the other patients’ psychotic symptoms were treated. |
| Schneider et al. [24] | A 40 year old male who at age 16 following a “flu-like illness” developed “behavioral disturbances with hypomania, insomnia, agitation, grandiose ideas, and pressure of speech”. While the specific medications trialed are not reported, it is noted that the patient developed EPS and became “akinetic-rigid” following treatment with “neuroleptic medication”. Lithium therapy reportedly “improved his psychological state”, and after it was discontinued due to hypothyroidism, he became more akinetic and mute. |
| Estiar et al. [25] | Three unrelated affected patients are described in the report. The first of whom was a 44 year old woman who at age 40 was observed laughing excessively with an inappropriate affect. At age 43 she was agitated as well as verbally and physically aggressive. While no frank psychotic symptoms are described, it is noted that she received haloperidol which may have contributed to her parkinsonism. The other two patients experienced frank psychotic symptoms but the use of antipsychotic medication is not described. |
| Odake et al. [26] | Three affected siblings are described in the report. The first patient described was a 56 year old female who developed paranoia at age 19 and was diagnosed with schizophrenia. While she was prescribed “several antipsychotics”, no further details in this respect are provided. At least one, and possibly both of her siblings, experienced psychotic symptoms but the use of antipsychotic medication is not described. |
| Satolli et al. [27] | A 51 year old female is described in the report. At the age of 20 she developed depression and delusions and has since been treated with olanzapine 10 mg/day. At some point thereafter she also experienced “complex visual hallucinations” and hypersexuality. She was also treated with levodopa/carbidopa 400 mg/day and ropinirole XL 8 mg/day. No information regarding her response to antipsychotic therapy is provided. |
